# Walking into the treatment room is not just a workflow: Therapeutic relationship as supportive care in daily radiotherapy

**DOI:** 10.1017/S1478951526103332

**Published:** 2026-07-23

**Authors:** Hsiao-Chuan Liu, Ching-Hsueh Cheng, Min-Chuan Chang, Yen-Ting Liu, Chao-Yuan Tu

**Affiliations:** Department of Oncology, National Taiwan University Hospital, Taiwan

In a previous Editorial, we argued that the time family members spend waiting outside the radiotherapy treatment room should not be seen as empty time outside care. After the patient enters the room, the family remains at the doorway, carrying repeated waiting, uncertainty, the work of receiving the patient afterward, and the daily task of bringing the treatment experience back home. That article brought the space outside the treatment room back into supportive care (Liu et al. 2026), echoing the reminder that caregiving is not merely informal accompaniment but work carried through the illness course (Applebaum 2022).

This Editorial takes one further step back. If waiting outside the door is not empty time, then the brief, ordinary, and hard-to-name interactions before and after a patient walks into the treatment room are not just workflow either. The earlier article applied this idea – that ordinary moments can themselves be care – to family caregivers. This one turns the same lens toward the relationship between the patient and the treatment team: another threshold moment, in which a patient may bring the bright parts of life into the treatment room.

In a radiotherapy department, the day often begins with schedules, positioning, image verification, calling patients in, machine time, and the next patient waiting. These tasks matter, and they must be precise. Yet after years of clinical practice, one slowly learns that the person walking into the room is never only a treatment site, a diagnosis, or the next fraction. Patients bring their bodies, but also fear, endurance, family, expectation, and whatever has happened in life that day.

An older patient was receiving radiotherapy. Because of his illness and treatment, speaking was difficult. His voice was sometimes unclear, and each expression took effort (details in this narrative have been altered to protect privacy). Each treatment day, we greeted him, asked how he was doing, and waited for him to answer at his own pace. These were not grand exchanges or formal psychosocial interventions. They were small habits: a greeting, eye contact, a little more time before the next step, and an attitude that did not rush him straight into the process.

One day, after treatment had finished, he was about to leave but suddenly stopped. He looked happy and, with effort, said, “I became a grandfather today.” A family member beside him smiled and added that they had only learned the news that morning. We were not reviewing images or handling a clinical problem. We simply shared his happiness and congratulated him.

It was too small a moment to appear in the medical record or become a quality indicator. Yet it reminded us that patients do not bring only suffering into the treatment room. Supportive and palliative care literature often speaks about distress, burden, grief, uncertainty, and caregiver suffering. These are important. But in daily treatment practice, patients also bring parts of life that remain bright and worth sharing. Such positive moments are rarely named, yet they may be evidence that a therapeutic relationship exists. That he stopped and worked hard to tell the team suggested that, for him, the treatment room was also a place where someone would listen, smile, and share it with him.

When we speak about the therapeutic relationship, we often think of clinical explanations, breaking bad news, treatment choices, prognosis discussions, or shared decision-making. These are important, and communication guidance emphasizes that patient-clinician communication supports trust, safety, and shared decisions (Gilligan et al. 2017). Recent work in *Palliative and Supportive Care* also reminds us that language and environment shape how patients and families receive difficult information (Miller et al. 2024). In radiotherapy, however, the therapeutic relationship is also formed in shorter, repeated, and less visible contacts. Patients lie on the same treatment couch each day, hear similar instructions, undergo similar immobilization, wait for the machine to move, and leave. For the system, this is a standardized workflow; for the patient, it may mean summoning courage anew each day.

If supportive care is placed only in formal assessment, consultation-room conversations, or referral pathways, the meaning of daily treatment contact can be missed. Radiation therapist-patient interactions influence experiences of information, support, comfort, and participation during treatment (Goldsworthy et al. 2023; O’Neill et al. 2023). Some patients may not remember every image match, but they may remember which therapist did not rush unclear speech, which nurse noticed more pain, which physician explained the treatment goal in language the family could understand, and which team still treated them as a whole person. This is consistent with dignity-conserving care: patients should not be defined only by disease but should still be seen, respected, and understood (Chochinov 2002). In serious illness, dignity and meaning are also preserved with family and caregiver participation (Sailian et al. 2025).

This is not a romantic view of clinical work. The treatment environment carries real pressure: high patient volume, limited machine time, tight workflows, low error tolerance, and emotional labor for frontline staff. Precisely because these pressures are real, brief, kind interactions should not be dismissed as decorative extras. They cannot replace adequate staffing, institutional support, psychosocial care, or palliative care teams. Nor do they ask radiation therapists, nurses, or other frontline staff to take on psychotherapy, family counseling, or medical decision-making beyond their professional boundaries. They are better understood as a small clinical space that allows patients to remain visible between pain and efficiency.

Supportive care here is also not one-way warmth from healthcare workers to patients. Clinical work has another side: patients sometimes give strength back to us. It may be a word of thanks, a nod, a look of relief after treatment, a family member saying “Thank you for your hard work,” or a patient bringing family good news into the room. These moments do not remove workload or replace reasonable systems. But they remind frontline staff that daily work is not only workflow; it is accompanying a person through a difficult stretch of life.

In this sense, kind interaction in the treatment room can be understood as a clinical loop. When frontline staff preserves a little real greeting and waiting, patients may be more willing to bring themselves into care. When patients feel safe, they may express discomfort or fear and may also share important life events. When the team hears these signals, care has a better chance of returning to the appropriate person. *Palliative and Supportive Care* has reminded us that patient and caregiver concerns should not merely be assumed but recognized and received (Oliveira et al. 2024). Brief interactions in daily radiotherapy are one place where such recognition can begin.

This loop does not require every treatment to become a deep conversation. In a busy department, several low-burden habits can be brought back into daily practice. First, a greeting should not be only a command or routine phrase; it should give the patient a brief moment of being seen. Second, when a patient speaks slowly, answers briefly, or looks hesitant, the team should not read this too quickly as an absence of need or smooth cooperation. Third, when a patient shares a worry or good news, a short, genuine response can hold the moment without turning it into counseling. Fourth, when these exchanges reveal pain, fear, family pressure, or difficulty understanding treatment, frontline staff should know how to share the signal with appropriate resources, such as physicians, nurses, social workers, dietitians, or palliative care teams.

That patient could have left quietly and kept the good news within his family. But he stopped. He wanted to tell the people who helped him complete treatment each day. That act of stopping was itself a form of trust. Cancer treatment did not become painless because of that moment. The next day, he might still have faced fatigue, discomfort, waiting, and treatment. But on that day, the treatment room held his joy. For us, it showed that patients do not bring only suffering into the room. They may also bring the parts of life that remain bright.

Previously, we looked from outside the treatment room and saw that family waiting can be part of supportive care. This Editorial points to an earlier and more ordinary starting point before the doorway: the patient walks into the room and is seen, waited for, and responded to. Daily radiotherapy is not only about enduring pain, completing fractions, and maintaining workflow. When the treatment team treats small moments as part of care, the treatment room can also become a place where patients and healthcare workers support one another and move forward together.

## Data Availability

Data sharing is not applicable because no datasets were generated or analyzed for this Editorial.
